# Pericardial effusions and cardiac tamponade in hospitalized systemic sclerosis patients: analysis of the national inpatient sample

**DOI:** 10.1186/s41927-023-00360-9

**Published:** 2023-09-28

**Authors:** Bikash Basyal, Waqas Ullah, Chris T. Derk

**Affiliations:** 1 Department of Medicine, Jefferson Abington Hospital, Abington, PA USA; 2 Division of Cardiology, Thomas Jefferson University Hospital, Philadelphia, PA USA; 3 Division of Rheumatology, University of Pennsylvania, 5th Floor White Bldg 3400 Spruce Street, Philadelphia, PA 19104 USA

**Keywords:** Systemic sclerosis, Scleroderma, Cardiac, Pericardial effusion, Tamponade

## Abstract

**Introduction:**

Clinically significant pericardial effusions and cardiac tamponade in systemic sclerosis (SSc) patients is uncommon and the factors that contribute to progression of pericardial involvement in SSc patients have not been well established.

**Methods:**

A review of the national inpatient sample database was performed looking SSc related hospitalizations between 2002 and 2019. Data was collected on patients with pericardial effusions and cardiac tamponade and analyzed to identify and describe patient characteristics and comorbidities.

**Results:**

Out of a total of 523,410 SSc hospitalizations, with an overall inpatient mortality rate of 4.7% (24,764 patients), pericardial effusion was identified in 3.1% of all hospitalizations (16,141 patients) out of which 0.2% (838 patients) had a diagnosis of cardiac tamponade. Patients with pericardial effusion were significantly more likely to have pulmonary circulatory disease (p = < 0.0001), congestive heart failure (p = < 0.0001) end stage renal disease (p = < 0.0001), diabetes (p = 0.015), and hypothyroidism (p = 0.025). Patients with cardiac tamponade were significantly more likely to have a history of coronary artery bypass graft surgery (p = 0.001) or atrial fibrillation (p = < 0.0001). Hospitalized patients with cardiac tamponade had a significantly increased mortality rate of 17.7% compared to 8.8% in patients with pericardial effusions without a tamponade physiology, with an odds ratio of 2.3 (1.97–2.86), p = < 0.0001.

**Conclusion:**

Pericardial effusion and tamponade are associated with increased morbidity and mortality in SSc patients. Further studies are required to explore the role of patient comorbidities and characteristics in development into pericardial effusions or tamponade.

## Introduction

Systemic sclerosis (SSc) is a chronic autoimmune rheumatic disorder that involves the skin and visceral organs characterized by an early inflammatory infiltrate, fibrosis, a vasculopathy, autoantibody production and is associated with significant morbidity and mortality. The most common cause of mortality is pulmonary and cardiac involvement. With advances in therapy as well as treatment of scleroderma renal crisis, hospitalization patterns and causes of mortality have changed over the years with cardiac and pulmonary involvement being associated with poor hospitalization outcomes [1]. The rates of inpatient mortality with SSc have been reported to be around 5–7% [1–3]. Prior studies from the National inpatient sample have also demonstrated economic impact with increased healthcare utilization and increased charges related to SSc hospitalizations [2, 3].

Cardiac involvement in SSc has been associated with up to 35% of cases and can involve valvular abnormalities, conduction abnormalities, coronary disease, myocardial or pericardial disease with associated increase in mortality [4]. Pericardial involvement causing large pericardial effusion and tamponade is infrequent but associated with increased mortality and poor outcomes in SSc patients [5]. A study from a single tertiary center with a dedicated SSc clinic has shown pericardial effusions to be present in 6.9% of SSc admissions [6], but there is limited data on patient comorbidities, disease progression, outcomes and treatment patterns.

In patients with SSc, pericardial effusions can be present in advanced SSc, but sometimes can be discovered early even preceding skin or other characteristic findings of SSc [7]. Large cohort studies have shown pericardial involvement is more frequent in diffuse cutaneous SSc (DcSSc) than in limited cutaneous SSc (LcSSc) subtype [8]. Autopsy series of SSc related deaths have shown presence of chronic pericarditis in more than two-thirds of cases and myocardial fibrosis in 37% of cases on histopathological examination [9] but it is not known how many of these patients had clinically significant pericardial disease. Much is yet to be known regarding predictors of pericardial effusion or cardiac tamponade and factors to be considered for prevention or treatment of this complication.

This study attempts to look at SSc related hospitalizations from an established national database and describe and identify associated comorbidities in SSc patients who have pericardial effusion or cardiac tamponade.

## Methods

This is a review of the National Inpatient Sample (NIS) database, which is a large national database allowing for assessment of hospital discharges across the country. A systematic search was made in the NIS database for the years 2002–2019, using standard international classification of diseases, clinical modification codes ICD-9-CM and ICD-CM-10. Informed consent and institutional review board approval were not required for this study given the public availability and completely de-identified nature data in the NIS database. SSc related hospitalizations were identified using the codes 710.1, 517.2, M34.0, M34.1, M34.2, M34.8, M34.81, M 34.82, M 34.83, M34.89, and M34.9. Data on comorbidities, and relevant baseline demographics including sex, hospital outcomes including mortality were collected. ICD-9-10 and ICD-10-CM codes for pericardial effusion (423.3, 423.9, I31.3, I31.4, I31.9) and cardiac tamponade (423.3, I31.4, I31.5, I31.6) were used to collect data on SSc patients. Relevant ICD codes were used to identify patient with different baseline characteristics, comorbidities and in hospital outcomes. [Table 1]

The comorbidities among SSc pericardial effusion and cardiac tamponade patients were compared using descriptive statistics. The Pearson chi-square test was used to compare the proportion of categorical values. The mean and standard deviation of normally distributed continuous variables were compared using the independent t-test analysis. The in-hospital outcomes for dichotomous variables were calculated using unadjusted Odds ratio on Cochran-Mantel-Haenszel test. The pooled estimates were presented with 95% confidence interval and an alpha criterion of p = < 0.05 was considered statistically significant.

A multivariate logistic regression analysis was performed to calculate the adjusted odds ratios accounting for potential confounders such as demographics and variable baseline comorbidities. All statistical analyses were performed with SPSS version 24.0.

## Results

During the period of 2002–2019 a total of 523,410 hospitalizations were identified in the NIS database with a diagnosis of SSc, with 84.3% being female patients, while 69.3% were identified as Caucasian which is similar to the demographics of the general SSc population. [Table 1] The average age at hospitalization was 58.49 years, with a standard deviation of 15.26. From this population, 183,958 (35.1%) of hospitalizations were reported to have coexisting pulmonary disease and 123,574 (23.64%) had pulmonary vascular disease listed as a comorbid condition. Congestive heart failure (CHF) and coronary artery disease (CAD) were reported to be present in 25.1% and 17.6% of all patients, respectively. During the period studied, 24,764 (4.7%) mortalities were recorded during the hospitalization.

**Table 1:** Characteristics and comorbidities of total SSc hospital admissions from 2002–2019

	N	Percentage
Sex		
Male	82,332	15.70%
Female	440,987	84.30%
Race		
White	316,298	69.30%
Black	70,859	15.50%
Hispanic	47,087	10.30%
Asian or Pacific islander	8068	1.80%
Native American	3631	0.80%
Other	10,681	2.30%
Pulmonary disease	183,958	35.1%
Pulmonary circulatory diseases	123,574	23.60%
Alcohol use	5456	1.00%
Anemia	45,369	8.70%
Hypothyroidism	106,719	20.40%
Obesity	12,760	2.40%
smoking	41,223	7.90%
Prior CABG	2271	0.4%
Congestive Heart failure	131,424	25.10%
Atrial fibrillation	76,072	14.50%
Coronary artery disease	91,988	17.60%
End stage renal disease	20,233	3.90%
Hypertension	277,780	53.10%
Diabetes	8940	1.70%
Died during hospitalization	24,764	4.7%

Pericardial effusion was identified in 3.1% of SSc hospitalizations (16,141 patients). A total of 838 patients (0.2% of total SSc patients) among the patients with SSc pericardial effusion had a diagnosis of cardiac tamponade. In terms of inpatient mortality, 17.7% (148 patients) with cardiac tamponade died during the hospitalization compared to 8.3% (1,268 patients) with a pericardial effusion without tamponade. Hospitalized SSc Patients with cardiac tamponade had a significantly increased mortality compared to hospitalized SSc patients with a pericardial effusion without tamponade physiology with an Odds ratio 2.3 (1.97–2.86), p = < 0.0001.

Compared to SSc patients with cardiac tamponade, SSc-pericardial effusion patients were significantly more likely to have pulmonary circulatory disease (p = < 0.0001) and congestive heart failure (p = < 0.0001). The presence of end stage renal disease (p = < 0.0001), diabetes (p = 0.015), and hypothyroidism (p = 0.025) was also more significantly present in the patients with pericardial effusion as compared to the tamponade patients. Similarly, the proportion of patients with hypertension (p = 0.667) and smoking history (p = 0.715) was also higher in patients with pericardial effusion as compared to patients with tamponade although this number was not statistically significant. [Table 2]

Patients with cardiac tamponade were significantly more likely to have had a history of atrial fibrillation (p = < 0.0001). The proportion of patients having coronary artery disease (p = 0.322) was also higher in patients with cardiac tamponade compared to SSc patients with pericardial effusion alone, although this was not statistically significant. [Table 2]

**Table 2:** Characteristics and comorbidities of Hospitalized SSc patients with Pericardial effusion and cardiac tamponade

			Pericardial effusion (N = 15,303)			Cardiac Tamponade (N = 838)	
	N	%		N	%		P value
In hospital mortality	1268	8.30%		148	17.70%		< 0.0001
Sex							
Male	2310	15.10%		158	18.90%		
Female	12,993	84.90%		680	81.10%		
Race							
White	7957	57.80%		422	54.80%		
Black	3459	25.10%		229	29.80%		
Hispanic	1616	11.70%		84	11.00%		
Asian or Pacific islander	303	2.20%		< 11	0.50%		
Native American	59	0.40%		< 11	0.60%		
Other	381	2.80%		25	3.30%		
Pulmonary disease	6814	44.5%		305	36.3%		< 0.0001
Pulmonary circulatory disease	7851	51.30%		306	36.50%		< 0.0001
Alcohol use	158	1.00%		< 11	0.00%		0.003
Anemia	2745	17.00%		155	18.50%		0.678
Hypothyroidism	3264	20.20%		144	17.20%		0.021
Obesity	365	2.40%		30	3.60%		0.029
Smoking	1130	7.40%		59	7.00%		0.715
Prior CABG	55	0.4%		< 11	1.2%		< 0.0001
Congestive Heart failure	7118	46.50%		302	36.00%		< 0.0001
Atrial fibrillation	2932	19.20%		203	24.30%		< 0.0001
Coronary artery disease	2328	15.20%		138	16.50%		0.322
End stage renal disease	1010	6.60%		25	3.00%		< 0.0001
Hypertension	8430	55.10%		455	54.30%		0.667
Diabetes	260	1.70%		< 11	0.60%		0.015

In patients with pericardial effusion, the presence of these comorbidities was independently associated with mortality. Patients with pulmonary circulatory diseases had increased mortality with adjusted Odds ratio of 2.75 (2.64–2.86, p < 0.0001). Similarly, there was increased mortality in patients with ESRD adjusted Odds ratio 1.34 (1.24–1.45, p < 0.001) and patients with CHF with adjusted Odds ratio 1.89 (1.82–1.96, p < 0.001).

In patients with cardiac tamponade, the presence of pulmonary circulatory disease and CHF similarly had increased mortality with adjusted Odds ratio of 1.55 (1.30–1.84, p < 0.001) and 1.34 (1.14–1.58, p < 0.001) respectively.

## Discussion

Pericardial involvement in systemic autoimmune diseases is thought to be related to the underlying systemic inflammation and may relate to a sudden flare or a gradual progression of the disease process. It is understood to be present in many rheumatologic diseases including systemic lupus erythematosus (SLE) and SSc, with more than 60% of cases reported to have pericardial involvement on histopathology of autopsy cases [10]. In patients with SLE, development of pleuritis, and size of pericardial effusions have been seen as predictors for development of tamponade, and high dose immunosuppressive treatment has been demonstrated to reduce need for surgical intervention [11].

The present study includes the largest cohort of hospitalized patients with SSc, from the NIS database from 2002 to 2019, with pericardial effusion noted in 3.1% of overall hospital admissions. The incidence of pericardial effusions in our study is lower than the one described in a single center trial which was at 6.9% of hospitalizations though this may relate to being a single tertiary referral center [6]. This is also likely to be an underestimate of true prevalence of pericardial effusions since echocardiograms may not get routinely performed in an inpatient basis and the data may not include respective ICD codes for pericardial effusions.

The patient population of this study were predominantly Caucasian females which is consistent with the overall demographics of SSc patients. Up to one-fifth of the hospitalized SSc patients had significant cardiac comorbidities such as congestive heart failure (25.1%) and pulmonary circulatory disease (23.6%), while pericardial effusion was clinically noted in 3.1% of this population of hospitalized patients, it is clearly lower than the 62–77% noted in autopsy series [9, 12], however it is understood that many patients may have only subclinical or not clinically apparent pericardial disease.

Compared to SSc patients with cardiac tamponade, SSc patients with a pericardial effusion were significantly more likely to have pulmonary circulatory disease and congestive heart failure. This may relate to the commonly subacute nature of SSc-PH which then leads to heart failure due to the progression of the underlying pulmonary hypertension. The association between PH and pericardial effusions has been clearly described in the general population as well as SSc patients. Unfortunately, the NIS database does not allow us to clearly identify which PH WHO classification group each of these patients was, though typically most SSc-PH patients are WHO Group 1 or Group 3, both typically subacute and slowly progressive.

The presence of diabetes and hypothyroidism was also more significantly present in patients with a pericardial effusion rather than cardiac tamponade, again the assumption is made that these are chronic slowly progressive illnesses and cardiac tamponade would more likely be an acute process. In the general population pericardial effusions have been described with hypothyroidism but not as commonly in association with diabetes. Similarly, the proportion of patients with end stage renal disease was also significantly higher in patients with pericardial effusion as compared to tamponade. Again, the NIS database does not allow for us to understand if these patients had SSc related kidney disease such as scleroderma renal crisis, or other forms of kidney disease.

Patients with cardiac tamponade on the other hand, were significantly more likely to have had a history of a prior coronary artery bypass graft (CABG), or atrial fibrillation. Some studies in the general population have suggested that a previous CABG protects against progression of a pericardial effusion to tamponade because of the presence of pericardial adhesions, though in this study it appears that this is not the case in SSc patients. The proportion of patients having coronary artery disease was also higher in patients with cardiac tamponade compared to SSc patients with pericardial effusion alone, although this was not statistically significant.

There are no specific treatment guidelines regarding treatment of pericardial effusions or tamponade in SSc patients. It can be assumed that patients with cardiac tamponade needed more emergent drainage or surgical intervention. We do not have information on treatment options or fluid characteristics on the NIS database, although prior studies have shown pericardial fluid to be exudative in nature [7].

Prior studies have showed significant burden of atrial fibrillation (AF) in rheumatic diseases and mortality, length of stay and hospitalization costs were also noted to be higher in patients with AF than patients without AF [13]. Atrial fibrillation in SSc can relate to causes such as seen in the general population which includes CAD, while in SSc patients there is a strong association with myocardial fibrosis. Our study also showed presence of AF in 16.5% of SSc patients with cardiac effusion and in 19% of SSc patients with cardiac tamponade, which is higher than the general prevalence of AF in SSc patients. Patients with AF were also significantly more likely to have pericardial tamponade than pericardial effusion, although it is difficult to establish direct causation or pathophysiologic relation with cardiac tamponade from this study.

There are some limitations of our study due to the nature of the data collected from the NIS. A major limitation is the lack of specific disease related data, including disease duration, disease subset (limited cutaneous SSc or diffuse cutaneous SSc), as well specific antibodies profile of patients or overlap with other autoimmune diseases. We do not have data on medications or laboratory values on the NIS database. A definite causal relationship of pericardial effusion or tamponade with the reported comorbidities or mortality cannot be established. In addition, pericardial effusion or tamponade being the immediate cause of death could not be ascertained. It would also be interesting to look at treatment including medical or surgical in patients with pericardial effusions and tamponade and its impact on patient outcomes.

## Conclusion

The presence of pericardial effusion and pericardial tamponade is associated with significant increase in in-hospital mortality in SSc- related hospitalizations. The development of pericardial tamponade is associated with significantly increased risk of in hospital mortality and may be associated with different patient comorbidities compared to pericardial effusions in SSc patients. It is important to identify and diagnose them in such patients to prevent overall morbidity and mortality. Further studies are required to explore the role and association of patient characteristics and comorbidities in development or progression into pericardial effusions or pericardial tamponade.

## Data Availability

The data supporting this study is available to a reasonable request to author Bikash Basyal ( Bikash.Basyal@jefferson.edu).

## References

[1] Chung L, Krishnan E, Chakravarty EF (2007). Hospitalizations and mortality in systemic sclerosis: results from the Nationwide Inpatient Sample. Rheumatology.

[2] Nietert PJ, Silverstein MD, Silver RM (2001). Hospital admissions, length of stay, charges, and in-hospital death among patients with systemic sclerosis. J Rhuematol.

[3] Ram Poudel D, George M, Dhital R, Karmacharya P, Sandorfi N, Derk CT (2018). Mortality, length of stay and cost of hospitalization among patients with systemic sclerosis: results from the National Inpatient Sample. Rheumatology.

[4] Kahan A, Coghlan G, McLaughlin V (2009). Cardiac complications of systemic sclerosis. Rheumatology.

[5] Hsu VM, Chung L, Hummers LK, Shah A, Simms R, Bolster M, Hant FN, Silver RM, Fischer A, Hinchcliff ME, Varga J, Goldberg AZ, Derk CT, Schiopu E, Khanna D, Shapiro LS, Domsic RT, Medsger T, Mayes MD, Furst D, Csuka ME, Molitor JA, Saketkoo LA, Salazar CR, Steen VD (2019). Risk factors for mortality and cardiopulmonary hospitalization in systemic sclerosis patients at risk for pulmonary hypertension, in the PHAROS Registry. J Rhuematol.

[6] Hosoya H, Derk CT. Clinically Symptomatic Pericardial Effusions in Hospitalized Systemic Sclerosis Patients: Demographics and Management. BioMed Research International 2018; 2018: 6812082. 10.1155/2018/6812082 PMC6008774 29967777

[7] Fernández Morales A, Iniesta N, Fernández-Codina A, Vaz de Cunha J, Pérez Romero T, Hurtado García R, Simeón-Aznar CP, Fonollosa V, Cervera R, Espinosa G (2017). Cardiac tamponade and severe pericardial effusion in systemic sclerosis: report of nine patients and review of the literature. Int J Rheum Dis.

[8] Fernández-Codina A, Simeón-Aznar CP, Pinal-Fernandez I, Rodríguez-Palomares J, Pizzi MN, Hidalgo CE, Del Castillo AG, Prado-Galbarro FJ, Sarria-Santamera A (2017). Fonollosa-Plà V and Vilardell-Tarrés M. Cardiac involvement in systemic sclerosis: differences between clinical subsets and influence on survival. Rheumatol Int.

[9] Byers RJ, Marshall DAS, Freemont AJ (1997). Pericardial involvement in systemic sclerosis. Ann Rheum Dis.

[10] Imazio M (2011). Pericardial involvement in systemic inflammatory diseases. Heart.

[11] Goswami RP, Sircar G, Ghosh A, Ghosh P (2017). Cardiac tamponade in systemic lupus erythematosus. QJM: An International Journal of Medicine.

[12] McWhorter JE, LeRoy EC (1974). Pericardial disease in scleroderma (systemic sclerosis). Am J Med.

[13] Khan MZ, Patel K, Patel KA, Doshi R, Shah V, Adalja D, Waqar Z, Franklin S, Gupta N, Gul MH, Jesani S, Kutalek S, Figueredo V (2021). Burden of atrial fibrillation in patients with rheumatic diseases. World J Clin Cases.

